# Structure, target-specificity and expression of *PN_LNC_N13*, a long non-coding RNA differentially expressed in apomictic and sexual *Paspalum notatum*

**DOI:** 10.1007/s11103-017-0679-4

**Published:** 2017-11-08

**Authors:** Ana Ochogavía, Giulio Galla, José Guillermo Seijo, Ana María González, Michele Bellucci, Fulvio Pupilli, Gianni Barcaccia, Emidio Albertini, Silvina Pessino

**Affiliations:** 1 Instituto de Investigaciones en Ciencias Agrarias de Rosario (IICAR-CONICET), Laboratorio de Biología Molecular, Facultad de Ciencias Agrarias, Universidad Nacional de Rosario, Parque Villarino, S2125ZAA Zavalla, Provincia De Santa Fe Argentina; 2 Laboratory of Genetics and Genomics, DAFNAE, University of Padova, Campus of Agripolis, Viale dell’Università 16, 35020 Legnaro, Italy; 3 Instituto de Botánica del Nordeste (IBONE-CONICET), Facultad de Ciencias Exactas y Naturales y Agrimensura, Facultad de Ciencias Agrarias, Universidad Nacional del Nordeste, Sargento Cabral 2131, 3400 Corrientes, Argentina; 4 Institute of Biosciences and Bioresources, Research Division of Perugia, National Research Council (CNR), via della Madonna Alta 130, 06128 Perugia, Italy; 5 Department of Agricultural, Food and Environmental Sciences, University of Perugia, Borgo XX Giugno 74, 06121 Perugia, Italy

**Keywords:** Apomixis, Apospory, LncRNA, *Paspalum*, Plant reproduction

## Abstract

**Key message:**

ncRNA PN_LNC_N13 shows contrasting expression in reproductive organs of sexual and apomictic *Paspalum notatum* genotypes.

**Abstract:**

Apomictic plants set genetically maternal seeds whose embryos derive by parthenogenesis from unreduced egg cells, giving rise to clonal offspring. Several *Paspalum notatum* apomixis related genes were identified in prior work by comparative transcriptome analyses. Here, one of these candidates (namely *N13*) was characterized. *N13* belongs to a *Paspalum* gene family including 30–60 members, of which at least eight are expressed at moderate levels in florets. The sequences of these genes show no functional ORFs, but include segments of different protein coding genes. Particularly, *N13* shows partial identity to maize gene BT068773 (*RESPONSE REGULATOR 6*). Secondary structure predictions as well as mature miRNA and target cleavage detection suggested that *N13* is not a miRNA precursor. Moreover, *N13* family members produce abundant 24-nucleotide small RNAs along extensive parts of their sequences. Surveys in the GREENC and CANTATA databases indicated similarity with plant long non-coding RNAs (lncRNAs) involved in splicing regulation; consequently, *N13* was renamed as *PN_LNC_N13*. The *Paspalum* BT068773 predicted ortholog (*N13TAR*) originates floral transcript variants shorter than the canonical maize isoform and with possible structural differences between the apomictic and sexual types. *PN_LNC_N13* is expressed only in apomictic plants and displays quantitative representation variation across reproductive developmental stages. However, *PN_LNC_N13-*like homologs and/or its derived sRNAs showed overall a higher representation in ovules of sexual plants at late premeiosis. Our results suggest the existence of a whole family of *N13*-like lncRNAs possibly involved in splicing regulation, with some members characterized by differential activity across reproductive types.

**Electronic supplementary material:**

The online version of this article (10.1007/s11103-017-0679-4) contains supplementary material, which is available to authorized users.

## Introduction

Apomictic plants form true seeds originating clonal offspring genetically identical to the mother (Nogler 1984). To that end, meiosis is avoided during the formation of gametes and embryos develop by parthenogenesis (Hand and Koltunow 2014). This trait is considered a deviation of sexuality, caused by genetic and/or epigenetic alterations affecting one or a few genes (Ozias-Akins 2006). It is often associated with polyploidy and/or hybridity, and displays a variety of mechanisms that evolved following a recurrent distribution pattern, suggesting a polyphyletic origin. The repeated emergence of numerous variants of the trait across evolution reinforces the idea that it might be governed by a few genetic determinants (Ozias-Akins 2006). Harnessing apomixis in agriculture would allow immediate fixation of any hybrid combination through an indefinite number of generations (Hand and Koltunow 2014). This capacity would dramatically reduce the efforts and costs inherent to plant breeding programs, as it was already shown for natural apomictic forage grasses (Ortiz et al. 2013). However, no major seed crops are apomictic, and attempts to introduce the trait from close relatives by cross-pollination have been unsuccessful to date. Therefore, the elucidation of the underlying molecular mechanisms of apomictic reproduction is essential for its effective use in breeding (Ortiz et al. 2013).

Sexual reproduction in flowering plants commonly involves the development of an embryo sac from the functional megaspore, the genetically reduced product that survives female meiosis (Hand and Koltunow 2014). Seed formation is typically achieved from mature embryo sac via double fertilization, a process in which the egg cell and the central cell polar nuclei fuse with sperm cells delivered by the pollen, to produce the zygote and the endosperm, respectively. In apomixis, two of the crucial steps of sexuality are avoided, i.e. meiosis and fertilization, in order to produce a progeny holding the same genetic constitution as the mother plant. The trait may alternatively follow two general mechanisms, known as sporophytic or gametophytic apomixis. In the first variant, which has been less characterized, supernumerary embryos are formed by somatic embryogenesis from nucellar cells surrounding the meiotic megagametophyte. The second possible pathway operates through the formation of one or several unreduced megagametophytes per ovule, in the absence of meiosis. Depending on the nature of the cell giving origin to the non-reduced embryo sacs, i.e. companion nucellar cells or the megaspore mother cell itself, apomixis is classified as aposporous or diplosporous, respectively. In gametophytic apomicts the endosperm can originate autonomously or following fertilization of the unreduced central cell (pseudogamy), depending on the species (Hand and Koltunow 2014).

*Paspalum notatum* Flügge, a rizomathous perennial grass native to Southern Mexico, Central America, the Caribbean and South America, is mainly represented in natural populations by two cytotypes with contrasting reproductive modes: allogamous sexual diploids (2n = 2x = 20) or self-fertile pseudogamous apomictic tetraploids (2n = 4x = 40) (Ortiz et al. 2013). During *P. notatum* apomictic development, one to several cells surrounding the megaspore mother cell (MMC) in the ovule nucellus achieve/s a gametic fate. After a series of mitosis, these ectopic apomeiotic spores give rise to supernumerary unreduced (2n) embryo sacs sometimes so numerous that take up the entire volume of the ovule. The egg cells belonging to the unreduced embryo sacs are able to initiate the formation of clonal embryos through parthenogenesis. In contrast, the central cell requires fertilization by the sperm cell in order to develop the endosperm (pseudogamy), but the requisite of a strict 2:1 maternal:paternal genome contribution ratio commonly observed in sexual individuals is somehow relaxed and can be unbalanced, as happens in many apomicts. In apomictic *P. notatum*, the endosperm formation is achieved in the presence of a 4:1 or several other maternal:paternal genome contribution ratios (Quarin 1999). This developmental alteration is useful to preserve functional endosperm formation after non-reduced polar nuclei fertilization by the meiotically-reduced sperm cell (Ortiz et al. 2013). Other apomictic species have evolved alternative mechanisms for successful endosperm formation, such as autonomous proliferation (*Hieracium* sp.) or fertilization of a single polar nucleus (*Eragrostis* sp). Multiple strategies to ensure seed viability have been discussed in reviews by Koltunow and Grossniklaus (2003) and Curtis and Grossniklaus (2008).

In a prior work aimed at identifying genes involved in apomictic development, we carried out a comparative analysis of the floral transcriptomes derived from artificially tetraploidized sexual genotypes and natural apomictic tetraploid genotypes of *P. notatum* (Laspina et al. 2008). Sixty-five transcript fragments showing differential expression among apomictic and sexual plants were identified, yet only 45 could be functionally annotated, while the remaining 20 showed no homology in the sequence databases. One of the identified transcripts (*N13*) revealed no coding potential, but included a short segment with similarity to a protein-coding plant gene. Based on this observation, we hypothesized that *N13* could represent a regulatory non coding RNA, whose activity might be involved in the switch between sexual and apomictic reproductive pathways (Laspina et al. 2008).

Non-coding RNAs (ncRNAs) integrate a group of heterogenous molecules that regulate gene expression at the transcriptional and post-transcriptional levels. They include small ncRNAs (sncRNAs, 20–30 nucleotide long, mainly miRNAs and siRNAs, commonly found as transcriptional and/or translational regulators), as well as medium and long ncRNAs (50–200 or > 200 nt, respectively), involved in splicing, miRNA target mimicry, gene inactivation and regulation of translation. These novel regulatory units have important roles in a wide range of biological processes, including the regulation of reproduction and the determination of sex (Li et al. 2015). In particular, lncRNAs are transcribed by RNA polymerase II or III, and additionally, by polymerase IV/V in plants. They are processed either by splicing or nonsplicing, polyadenylation or non-polyadenylation, and can be located in the nucleus or the cytoplasm. Unlike miRNAs, only a small set of lncRNAs is known to function in different developmental processes. They operate through diverse mechanisms, such as forming modular scaffolds in polycomb-mediated repression, activating chromatin-remodeling complexes and attenuating miRNA- mediated repression through miRNA target mimicry. Among these regulatory mechanisms, lncRNAs regulate numerous developmental processes by associating with sncRNAs (Ariel et al. 2015).

In this work we used full genome surveys, analysis of next generation sequencing (NGS) databases revealing the long and small RNA fractions of the sexual and apomictic floral transcriptomes, secondary structure folding and mature miRNA predictions, target cleavage experiments, qPCR expression measurements and in situ hybridization to investigate the structure, expression and putative activity of the non-coding RNA *N13*, previously reported as differentially expressed in reproductive organs of apomictic and sexual *P. notatum* (Laspina et al. 2008). Based on our results, we propose a role for *N13* as a member of a whole family of long non-coding RNAs, which might be modulating particular targets in order to drive the reproductive strategy.

## Materials and methods

### Plant material

The following genotypes were used: C4-4x: sexual tetraploid derived from the duplication of diploid plant C4-2x  (2n = 4x = 40) (Quarin et al. 2001); Q4188: sexual tetraploid derived from the cross Q3664 × Q3853 (in turn, parent Q3664 originated from a cross between the sexual tetraploid plant PT-2, induced by colchicine treatment of the sexual diploid Pensacola bahiagrass biotype, *P. notatum* var. *saurae*, and the white-stigma bahiagrass strain WSB) (2n = 4x = 40) (Quarin et al. 2003); Q4117 highly apomictic natural tetraploid accession from Brazil (2n = 4x = 40) (Ortiz et al. 1997); R1, an odd facultative aposporous diploid genotype, able to occasionally form non-reduced embryo sacs, derived from sexual diploid accession Q4084, native to Cayastá, Santa Fe, Argentina (2n = 2x = 20) (Quarin et al. 2001). The developmental stage at which spikelets were collected was evaluated by analyzing the macromorphology of inflorescences and the stage of pollen development, following the methods and the reproductive calendar reported by Laspina et al. (2008).

### Genome survey

Genomic DNA was extracted from young leaves using the CTAB method (Saghai-Maroof et al. 1984). PCR reactions were performed in a final volume of 25 μL including 60 ng DNA, 0.2 μM specific primers, 1x PCR buffer (Promega), 2.5 mM MgCl_2,_ 200 μM dNTPs and 1.5 U of Taq Polymerase (Promega). Primers used are shown in Table 1. Cycling was done in an MJ Research thermocycler and consisted of the following steps: 5 min at 94 °C, 40 cycles of 30 s at 94 °C, 1 min at 58 °C and 1 min at 72 °C. A final elongation of 10 min at72 °C was also included. Amplified fragments were loaded onto 5% polyacrylamide gels, electrophoresed in TBE 1X buffer and silver stained. Genomic sequences corresponding to the whole *N13* family were recovered from a *P. notatum* genomic sequence raw data library (see “Materials and methods”, NGS data libraries) currently available in our laboratory (unpublished). Raw reads showing similarity to *N13* were assembled into contigs by using the EGAssembler online tool (http://www.genome.jp/tools/egassembler/) (Masoudi-Nejad et al. 2006). Contigs and singletons were aligned with Clustal Omega (http://www.ebi.ac.uk/Tools/msa/clustalo/). The alignment file was submitted to Simple Phylogeny (http://www.ebi.ac.uk/Tools/phylogeny/simple_phylogeny/) to produce a phylogenetic tree revealing the degree of relatedness among different sequences.

**Table 1:** Primers used for genomic amplification, 5′-3′ RACE, qPCR and N13Tar cleavage detection experiments

Experiment	Primer	Sequence
N13 GENOMIC AMP	G13-upper	5′-GCCCTTAGCCATTTACACTTATTTAG-3′
N13 GENOMIC AMP	G13-lower	5′-TATCTTCACTCGACTTGTACCGTTTAGG-3′
5′-3′ RACE	A-13-upper1	5′-GCCCTTAGCCATTTACACTTATTTAG-3′
5′-3′ RACE	A-13-upper2	5′-GGAGTTCCACCACCCTATATTTGTTTG-3
5′-3′ RACE	A-13-lower1	5′-TATCAGGGGTTTATGCATGTGGGGTTTC-3′
5′-3′ RACE	A-13-lower2	5′-TATCTTCACTCGACTTGTACCGTTTAGG-3′
5′-3′ RACE	AP1	5′-CCATCCTAATACGACTCACTATAGGGC-3′
5′-3′ RACE	AP2	5′-ACTCACTATAGGGCTCGAGCGGC-3′
qPCR	A-13-upper2	5′-GGAGTTCCACCACCCTATATTTGTTTG-3′
qPCR	A-13-lower2	5′-TATCTTCACTCGACTTGTACCGTTTAGG-3′
qPCR	Tar-N13 upper	5′-CAAGCCAAAGCACACACACCTC-3′
qPCR	Tar-N13 lower	5′-GATCAGGTTCACAGCCACATCCT-3′
qPCR	G6PdH upper	5′-TGAATCTAGTCCATCCGCTTG-3′
qPCR	G6PdH lower	5′-TCATCAGGCAGGGAAGCTA-3′
qPCR	Tub upper	5′-GTGGAGTGGATCCCCAACAA-3′
qPCR	Tub lower	5′-AAAGCCTTCCTCCTGAACATGG-3′
CLEAVAGE	Tar-N13 low1	5′-GCCGCACAGGTTTTAGGAAGAAC-3′
CLEAVAGE	Tar-N13 low2	5′-CCTTCCTCCAGGCACCTATTGAT-3′
CLEAVAGE	Tar-N13 low3	5′-GATCAGGTTCACAGCCACATCCT-3′

### NGS data libraries

454/Roche FLX + transcriptome libraries were produced in prior work (Ortiz et al. 2017) by our research group and Instituto de Agrobiotecnología de Rosario, Argentina (INDEAR), from spikelets of plants Q4117 and C4-4x (see “Plant material”). The 454/Roche FLX + sequence datasets supporting the conclusions of this article are publicly available at the NCBI Sequence Read Archive (SRA) repository, https://www.ncbi.nlm.nih.gov//bioproject/PRJNA330955, under accession numbers SRX1971037 and SRX1971038 for apomictic (Q4117) and sexual (C4-4x) libraries, respectively. The Transcriptome Shotgun Assembly (TSA) projects corresponding to the apomictic (Q4117) and sexual (C4-4x) samples have been deposited at DDBJ/ENA/GenBank under the accessions GFMI00000000 and GFNR00000000, respectively. The versions described in this paper are the first ones (GFMI02000000 and GFNR01000000, respectively). Triplicate Illumina sRNA libraries (3 apomictic and 3 sexual) were produced in a prior work from the same floral samples. The miRNA bioinformatics analysis was carried out at Instituto de Agrobiotecnología de Rosario, Argentina (INDEAR). The sRNA datasets supporting the conclusions of this article are available in the NCBI Sequence Read Archive (SRA) repository under accession number SRP099144. To construct both the 454/Roche FLX + and the Illumina small RNA libraries, spikelets collected at premeiosis, meiosis, postmeiosis and anthesis were equitably mixed and used for RNA extraction. The selection of the material was done by following the reproductive calendar published by Laspina et al. (2008). The *Paspalum simplex* Illumina LMC (laser microdissected cells) sequence database was provided by Galla, Bellucci, Barcaccia and Pupilli (unpublished). The *P. notatum* genomic raw data library (unpublished) was constructed from DNA extracted from leaves of genotype R1, a facultative aposporous diploid genotype (occasionally able to form non-reduced embryo sacs), derived from sexual diploid accession Q4084, native to Cayastá, Santa Fe, Argentina (2n = 2x = 20) (Quarin et al. 2001). Sequencing was conducted at Instituto de Agrobiotecnología de Rosario, Argentina (INDEAR).

### Rapid amplification of cDNA ends (RACE)

Marathon non-cloned cDNA library (BD Biosciences Clontech) were prepared from total RNA obtained from Q4117 and Q4188 spikelets at late pre-meiotic developmental stage I, following the *P. notatum* reproductive calendar reported by Laspina et al. (2008). This stage is immediately preceding the onset of aposporous initials. Total RNA was isolated from inflorescences using the SV Total RNA Isolation System (Promega). Poly(A) + RNA was purified from RNA samples using Dynabeads (Dynal^®^), according to manufactures’ protocol. Poly(A) + RNA was reverse-transcribed, double-strand cDNA was synthesized and linked to Marathon cDNA adaptors following the Marathon cDNA amplification kit (BD Biosciences Clontech) protocol. RACE experiments were carried out following the recommendations of the Marathon cDNA amplification kit manufacturers (BD Biosciences Clontech). To amplify the 5′ and 3′ flanking regions, two pairs of nested reverse-oriented gene-specific primers were designed for the candidate sequence (Table 1). To amplify *N13TAR* cleavage products, a set of three nested primers located downstream to the predicted cleavage site were used, in combination with adaptor-specific primers (Table 1). Each target was amplified by duplicate from both libraries, using specific primers and the Marathon AP2 adapter in three successive rounds (from the most external specific primer to the most internal one). The fragments were separated in polyacrylamide gels, revealed with silver staining, cloned and sequenced. In all cases, the RACE oligonucleotides had 50–70% GC content with a melting temperature ≥ 67 °C. PCR reactions were prepared in a 50 μL final volume containing 2 μL of Marathon library product (BD Biosciences Clontech), 1X GoTaq activity buffer (Promega), 200 μM dNTPs, 0.2 μM of the gene-specific primer, 0.2 μM of adaptor-specific primer (AP1 or AP2) and 1.5 U of GoTaq DNA polymerase enzyme (Promega). Initial PCR conditions were the following: 94 °C for 1 min followed by 30 cycles of 30 s at 94 °C and 4 min at 68 °C (both annealing and polymerization temperatures were 68 °C). Several PCR rounds (2–4) were performed. Cycling temperatures were optimized according to the Tm of the particular set of primers used. Positive and negative controls were included. Positive controls consisted of amplifications with two specific oligonucleotides matching in opposite sense, which amplified a small segment within the original sequence fragment. Negative controls consisted of amplification reactions using specific and adapter-complementary primers in the absence of template DNA. After examination in agarose gels (5′ and 3′ extension) or polyacrylamide gel electrophoresis (target detection), the products were isolated by using the SV Wizard Gel and PCR Clean up System (Promega) or eluted in sterile distilled water and re-precipitated, respectively. Amplicons were cloned into pGeM-T easy (Promega) vector and transformed into *E. coli* by electroporation. Recombinant plasmids were purified with Wizard Plus SV Minipreps (Promega) and insert verification was carried out by PCR using the M13 forward and reverse primers and the following amplification conditions: 94 °C for 1 min, 25 cycles of 94 °C for 30 s, 55 °C to 68 °C for 1 s, 72 °C for 1 min. Sequencing was performed at Macrogen Inc (Korea). Both strands were sequenced for several candidates by using M13 forward and reverse primers, in order to control sequencing quality.

### Bioinformatics analysis

Vector contaminations were identified and removed by using the VecScreen tool at the National Center for Biotechnology Information (NCBI) webpage (http://www.ncbi.nlm.nih.gov/VecScreen/). Aligments were done with Clustal Omega on the EBI-EMBL website (http://www.ebi.ac.uk/Tools/msa/clustalo/). Neighbour-joining phylogenetic trees without distance corrections were produced at the Clustal Omega website (http://www.ebi.ac.uk/Tools/msa/clustalo/) or Simple Phylogeny (http://www.ebi.ac.uk/Tools/phylogeny/simple_phylogeny/). DNA assemblies were done with the EGAssembler tool (http://www.genome.jp/tools/egassembler/) (Masoudi-Nejad et al. 2006). Analysis of DNA similarity was carried out using the BLASTn and BLASTx packages at NCBI (http://www.ncbi.nlm. nih.gov/BLAST/), the *Arabidopsis* Information Resource website (http://www.arabidopsis.org/blast/) and the Gramene website (http://www.gramene.org). BLAST analyses on the 454 and the sRNA databases were carried out at the INDEAR webservices’ site. BLASTn analyses on the LMC libraries were performed by using version v2.2.31 + of BLAST (ftp://ftp.ncbi.nlm.nih.gov/blast/executables/blast+/) with default parameters. Open reading frames were searched with the ORF finder at NCBI (http://www.ncbi.nlm.nih.gov/projects/gorf/). Secondary structure folding predictions were made in the Mfold 2.3 from the Mfold Web Server (http://unafold.rna.albany.edu/?q=mfold). Target detection was carried out with psRNATarget (http://plantgrn.noble.org/psRNATarget/) (Dai and Zhao 2011). Hybridization stability between the miRNAs and their putative targets were analyzed with RNAhybrid software (Rehmsmeier et al. 2004). LncRNA blast analysis was done on the GreenC (Paytuví Gallart et al. 2016), the CANTATA (Szcześniak et al. 2016) and the NONCODE (Bu et al. 2012) lncRNA databases. For sRNA mapping, the Bowtie v. 1.1.2 program (bowtie-bio.sourceforge.net) was used to align reads against the reference transcriptomes, by allowing 0 mismatches (− n), requiring 19 high quality bases to initiate the alignment (− l) and selecting the option ‘best’ for report of the best alignments only. For read counting, the program Subread v 1.5.0-p1 (module feature counts) was used (http://subread.sourceforge.net/), with the Q parameter set as 10 and no strand selection. For transcriptome differential representation analysis the software EdgeR (https://bioconductor.org/packages/release/bioc/html/edgeR.html) was used, with the parameter normethod (normalization method) set at upper quartile and a cpm value = 2.

### Real-time PCR experiments

Total RNA was extracted from spikelets collected at early pre-meiosis (stage 0), late pre-meiosis/meiosis (stage I/II), post-meiosis (stage III/IV/V/VI) and anthesis (stage VII) (Laspina et al. 2008), by using the SV Total RNA Isolation kit (Promega). The derived cDNA was synthetized with Superscript II (Invitrogen) and a polyT-anchored primer. PCR primer pairs were designed by using Primer 3 (http://biotools.umassmed.edu/bioapps/primer3_www.cgi) (Table 1). Oligonucleotides were synthesized by IDT (Integrated DNA technologies, http://www.idtdna.com/Home/Home.aspx). RT-PCR reactions were prepared in a final volume of 25 µL containing 200 nM gene specific primers, 1X Mezcla Real qPCR (Biodynamics) and 20 ng of reverse-transcribed RNA (produced with *Superscript* II, Invitrogen-Life Technologies). Beta-tubulin and glucose-6-phosphate were used as reference genes, as it was recommended by other authors working in the same plant system (Ochogavía et al. 2011; Felitti et al. 2011; Podio et al. 2014). RT (−) and non-template controls were incorporated to the assays. Two biological replicates were individually processed in three technical repeats. Amplifications were performed in a Rotor-Gene Q thermocycler (Qiagen), programmed as follows: 2 min at 94 °C, 45 cycles of 15 s at 94 °C, 30 s at 63 °C, 40 s at 72 °C. A melting curve (10 s cycles from 72 to 95 °C, where temperature was increased by 0.2 °C after cycle 2) was produced at the end of the cycling to check consistent amplification of a single amplicon. Relative quantitative expression was estimated by using REST-RG (Relative Expression Software Tool V 2.0.7 for Rotor Gene, Corbett Life Sciences), considering the take-off values and amplification factors for each particular reaction. We selected the sample showing the lowest transcript representation as relative control (for this sample the value of expression was arbitrarily assigned as 1) in order to display relative expression values > 1. *N13* qPCR products were run in agarose gels 1.5% and cloned in pGeM-T easy (Promega) as indicated above (see RACE section). Eight apomictic and eight sexual clones were sequenced by Macrogen Inc (Korea).

### In situ hybridization (ISH) analyses

ISH experiments were performed on Q4117 and C4-4x spikelets collected at premeiosis/meiosis. Flowers were dissected, fixed in 4% paraformaldehyde/0.25% glutaraldehyde/0.01 M phosphate buffer pH 7.2, dehydrated in an ethanol series and embedded in paraffin. Specimens were cut into sections of 10 μm and placed onto slides treated with poly-L-lysine 100 μg/ml. The paraffin was removed with a xylene series. The plasmid including the original *N13* fragment isolated by Laspina et al. (2008) was linearized using restriction enzymes *Nco*I or *Sal*I (Promega). The probe was labeled with the Roche Dig RNA Labeling kit (SP6/T7), following the manufacturers’ instructions. The template digested with *Sal*I or *Nco*I restriction enzymes was used to produce a probe from the T7 (+) or SP6 (−) transcription starts, respectively. Probes were hydrolyzed to 150–200 bp fragments. Prehybridization was carried out in a buffer of 0.05 M Tris–HCl pH 7.5, containing 1 μg/mL proteinase K in a humid chamber at 37 °C for 10 min. Hybridization was carried out overnight in a humid chamber at 42 °C, in buffer containing 10 mM Tris–HCl pH 7.5, 300 mM NaCl, 50% formamide (deionized), 1 mM EDTA pH 8, 1 X Denhardt, 10% dextransulphate, 600 ng/mL total RNA and 60 ng of probe. Detection was performed following the instructions of the Roche Dig Detection kit, using anti DIG AP and NBT/BCIP as substrates. After antibody reaction, detection, and washing, sections were mounted in glycerol 50% and observed under a light microscope.

## Results

### *N13* genomic representation

*N13* was originally identified by Laspina et al. (2008) as a transcript-derived cDNA fragment of 532 nucleotides, whose expression was upregulated in apomictic genotypes of *P. notatum* at premeiosis/meiosis (sequence available at GenBank under accession number KX900513). Since its sequence showed no homology with known gene products in NCBI surveys, no putative function could be assigned. Therefore, we decided to conduct further studies in order to characterize its structure, expression and specific activity during apomictic development. Firstly, the occurrence of the sequence in the genome was analyzed in sexual and apomictic *Paspalum* genotypes. PCR amplifications were carried out on genomic DNA originated from plants Q4188 and Q4117 using *N13* specific primers (Table 1). Three major amplicons were produced, out of which one was present only in the sexual genotype (Supplementary Online Resource 1, left panel). The emergence of several amplicons suggested the existence of more than one allele or gene/paralog for this sequence. Then, we used a *P. notatum* full genome raw data library available in our laboratory (unpublished) to study the global representation of *N13* homologs. A total of 835 sequences (250 nt long) similar to *N13* (E-val < e^−10^) were detected. The assembly of these sequences revealed 60 unisequences (47 contigs and 13 singletons), indicating the existence of at least 60 different homologs (alleles or gene/paralogs) (E-values ranging from 0.0 to 2 e^−76^; %ID ranging from 98 to 82%). *N13* resulted included in a contig formed by 14 reads, showing high similarity with the original sequence (E-val: 0.0; ID: 98%; coverage: 532 nucleotides). These results indicate that *N13* is a *Paspalum* genomic non-coding sequence and belongs to a gene family including from 30 to 60 members.

### *N13* expression in florets of sexual and apomictic genotypes

Next, we used 454/Roche FLX + floral transcriptome libraries of sexual and apomictic *P. notatum* available in our lab to detect sequences related with the *N13* family expressed in florets at developmental stages spanning from premeiosis to anthesis. The database accession numbers and similarity parameters of all detected isotigs are summarized in Table 2. In apomictic plants, three highly similar isotigs were identified (apoisotig00494, apoisotig00492, apoisotig00490). In sexual plants, related sequences were also detected, but they scored lower coverage and less similarity (sexisotig01384 and sexisotig01380) (Table 2). Using all contigs and singletons detected in the genome as queries, additional homologous isotigs displaying lower similarity values were detected in flowers: apoisotig03642; apoisotig15855; apoisotig39851; apoisotig21541; sexisotig33073; sexisotig32142; sexisotig20966; sexisotig09727; sexisotig33916; sexisotig09642; sexisotig09641 and sexisotig05155 (Table 2). The sequence lengths vary from 529 to 3783 nucleotides, revealing a large structural diversity. They were found expressed at moderate levels (number of reads ranging from 0 to 6, depending on the library), hampering a preliminary prediction of differential expression between sexual and apomictic genotypes.

**Table 2:** *Paspalum notatum* floral transcripts showing similarity to *N13*

Isotig	GenBank accession^a^	Isogroup	Query cover^b^ (%)	E value	Identity (%)
apoisotig00494	GFMI02000550.1	apoisogroup00028	99	7 e^−125^	80
apoisotig00492	GFMI02000548.1		99	7 e^−125^	80
apoisotig00490	GFMI02000546.1		99	7 e^−125^	80
apoisotig03642	GFMI02003695.1	apoisogroup00484	31	3 e^−46^	86
apoisotig15855	GFMI02015904.1	apoisogroup05727	30	2 e^−43^	86
apoisotig39851	GFMI02039881.1	apoisogroup29406	27	5 e^−38^	86
apoisotig21541	GFMI02021592.1	apoisogroup11096	25	9 e^−35^	86
sexisotig01384	GFNR01001404.1	sexisogroup00104	83	5 e^−95^	79
sexisotig01380	GFNR01001400.1		83	5 e^−95^	79
sexisotig33073	GFNR01033088.1	sexisogroup24615	27	9 e^−41^	86
sexisotig32142	GFNR01032157.1	sexisogroup23684	32	3 e^−46^	85
sexisotig20966	GFNR01020985.1	sexisogroup12508	27	2 e^−43^	88
sexisotig09727	GFNR01009747.1	sexisogroup03099	27	2 e^−36^	84
sexisotig33916	GFNR01033930.1	sexisogroup25458	27	2 e^−36^	84
sexisotig09642	GFNR01009662.1	sexisogroup03056	27	5 e^−38^	84
sexisotig09641	GFNR01009661.1		27	5 e^−38^	85
sexisotig05155	GFNR01005180.1	sexisogroup00970	26	1 e^−38^	87

Sequences showing the highest similarity in the apomictic and sexual plant were underlined

^a^The Transcriptome Shotgun Assembly (TSA) projects corresponding to the apomictic (Q4117) and sexual (C4-4x) samples have been deposited at DDBJ/ENA/GenBank under the accessions GFMI00000000 and GFNR00000000, respectively. The versions described in this paper are the first ones (GFMI02000000 and GFNR01000000, respectively)

^b^Query: N13 original sequence

To confirm expression in *Paspalum* reproductive organs, we carried out a survey in the only *Illumina* transcriptome library of ovules currently available for the genus *Paspalum*, which was constructed from laser microdissected nucellar cells of sexual and apomictic *P. simplex* genotypes at late premeiosis (Galla, Barcaccia, Bellucci and Pupilli, unpublished). Five sequences were similar to *N13* with E-values < e^−10^. Other 19 sequences showed E-values in the range e^−10^– 0.001. The best hits were scored by transcripts 420632/578620 and 420633/578620, whose sequence length was 336 and 371 nucleotides, respectivey, and matched with IDs of 82% (E-value: 3e^−39^) and 80% (E-value: 4e^−37^), respectively. These results confirm that sequence *N13* is expressed in flowers of apomictic *Paspalum* together with other members of its family. In apomictic plants, sequences highly similar to the original *N13* clone (Laspina et al. 2008) were detected, whereas less similar members were found in sexual plants.

### *N13* extension of cDNA ends

RACE (Rapid Amplification of cDNA Ends) (Chenchick et al. 1996) experiments were performed in order to extend both 5′ and 3′ ends on apomictic and sexual *P. notatum* floral non-cloned Marathon libraries. A PCR product was produced only from 5′ amplifications of the apomictic plant (Supplementary Online Resource 1, panel right). Isolation, cloning and sequencing revealed several sequences of slightly different sizes which overlapped with the original transcript. While this observation confirmed the presence of *N13*-related sequences in the transcriptome, further extension of the original sequence was not achieved.

### *N13* annotation

Computational analysis showed no predicted ORFs of relevant size in any frame. *N13* and its related family members showed no homology in NCBI, TAIR and Gramene BLASTN and BLASTX surveys. Here again, the existence of very short conserved fragments was observed for several members of the family. In the case of the original fragment *N13*, 100% identity with the *Zea mays* gene BT068773 (encoding RESPONSE REGULATOR 6) was detected along 22 nucleotides (*N13*: 405-426; BT068773: 45-66). This region is included in a larger one showing partial similarity with the same gene along 71 nt (*N13*: 359-430; BT068773: 35-69). Moreover, the same of *N13* sequence potentially recognizes a second location within BT068773 (*N13*: 414-430; BT068773: 285-300). Other members of the *N13* family expressed in flowers showed the same type of partial similarity with the following protein-coding transcripts: unknown ZM_BFb0088N15 of *Zea mays* (apoisotig00494, GenBank accession number GFMI02000550.1); F-box domain containing protein XM_008674592 of *Zea mays* (apoisotig00492, GenBank accession number: GFMI02000548.1); dead-box ATP-dependent RNA helicase 52C XM_015762339 of *Oryza sativa* (apoisotig00490, GenBank accession number: GFMI02000546.1); unknown ZM_BFb0046G14 of *Zea mays* (sexisotig01384, GenBank accession number: GFNR01001404.1) and peptidyl-prolyl isomerase FKBP12 EU959401 of *Zea mays* (sexisotig01380, GenBank accession number: GFNR01001400.1).

### Target prediction

An analysis using psRNATarget and selecting the *Zea mays* DFCI Gene Index as database for target search predicted the accession TC562477 (Response Regulator 6) as putative target, a gene highly similar to BT068773 (E-value: 0; identity 766/788, 99%; Gaps: 0/788, 0%). Therefore, we hypothesized that *N13* was a regulatory non-coding RNA, whose putative target was the *P. notatum* ortholog to BT068773 (from now on called *N13TAR*). To verify this hypothesis, the possible function of *N13* as miRNA precursor was analyzed. We searched the predicted mature miRNAs in the *P. notatum* floral sRNA libraries and analyzed the cleavage of the putative target at the predicted site, between nucleotides 10 and 11 of the sequence matching the mature miRNA, mediated by PAZ-PIWI ARGONAUTE (Rhoades et al. 2002) (see below).

### *N13* is not a miRNA precursor

**Fig. 1:**
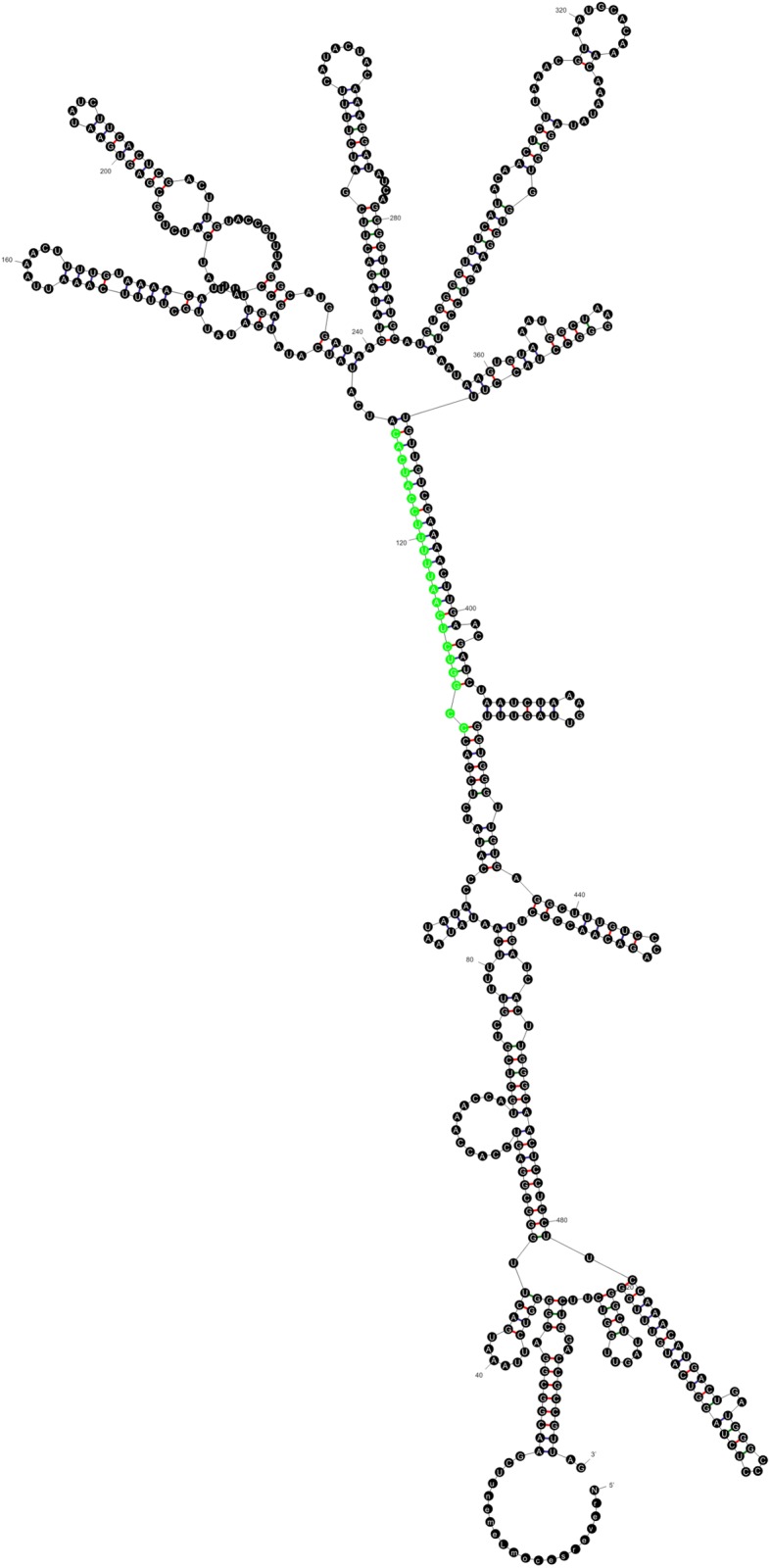
Secondary structure prediction. Folding prediction corresponding to *N13* (−) strand. The green portion located in a stem corresponds to the conserved sector homologous to BT068773. Distinctive bubbles usually present in plants pre-miRNAs are absent

**Fig. 2:**
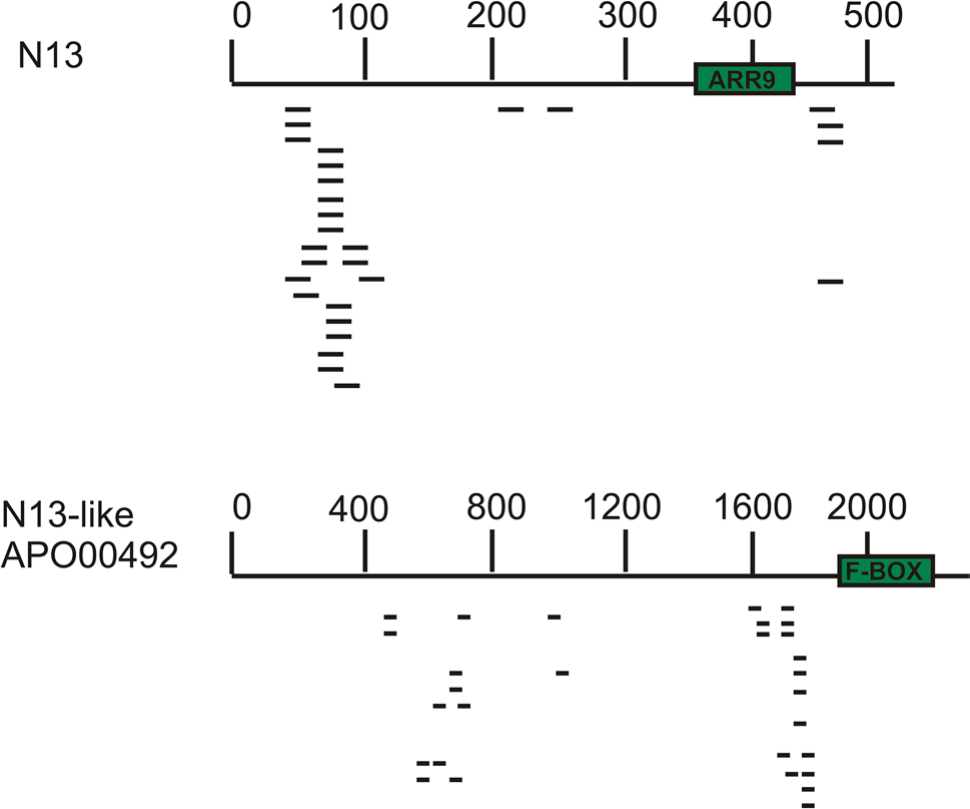
Small RNA originated from *N13* family members. Small RNAs originated from the apomictic sRNA libraries mapped on the original N13 sequence (top panel) or the apoisotig00492 N13-like sequence (bottom panel). The predicted BT068773 (ARR9) (top) and F-BOX (bottom) recognition sites were marked in green. sRNAs are located in regions different from the predicted target recognition site

The *N13* most stable predicted secondary structure corresponded to the (−) strand (dG= − 173.98) (Fig. 1). Although the BT068773 conserved portion was located in a stem, the characteristic bubbles present in miRNAs (Schwab et al. 2005) were not observed and the dimensions did not adjust to the reported parameters (Fig. 1). Northern blots and PCR amplifications failed at detecting the presence of a mature miRNA in RNA samples extracted from florets of sexual and apomictic plants (data not shown). Then, we conducted BLAST searches onto Illumina small RNA data libraries generated in prior work by our research group (GenBank accession number SRP099144). No small RNAs were associated with the putative target site (359-430), but the presence of numerous small RNAs distributed in different parts of the *N13* sequence was detected (Fig. 2, top panel). These sRNAs were 24-nucleotide long, displayed both orientations (plus and minus), and exhibited higher representation in the 1–100 and 430–500 regions. The rest of the *N13* precursor family members present in the 454/Roche FLX + libraries also revealed association with a large number of 24-nucleotide long sRNA, which were located out of the predicted target sites. This profile was schematized for a representative member of the family (*N13*-like apo00492) (Fig. 2, bottom panel). Moreover, 5′ RACE experiments of *N13TAR* were conducted on two non-cloned Marathon libraries (Clontech) originated from flowers of the apomictic and the sexual genotypes at late premeiotic/meiotic stage. These experiments were aimed at confirming or discarding the ARGONAUTE-mediated activity of a putative *N13* mature miRNAs (i.e. cleavage at the ARGONAUTE predicted site of processing) onto the target. Only the sexual Marathon library produced amplification fragments. However, after cloning and sequencing, the segments did not match the predicted target sequence, but other sequences displaying low similarity, and none of them ended in a sequence similar to the cleavage predicted site. Altogether, the experimental evidence indicated that *N13* is not a miRNA precursor, but possibly a different type of ncRNA. *N13* neither forms a mature miRNA from its predicted recognition site nor induces the cleavage of its putative target at the site of recognition. Family members might be rather acting through an alternative mechanism, probably mediated by 24-nucleotide sRNAs.

### Representation of *N13* small RNA sequences in florets of sexual and apomictic plants

As mentioned above, members of the *N13* family produce abundant 24-nucleotide sRNAs. To analyze if they were differentially represented in the sexual and apomictic biotypes, the small reads occurring in the sexual and apomictic Illumina sRNA libraries were mapped onto a global assembly of the 454/Roche FLX + libraries (constructed by combining apomictic + sexual long read sequences). The number of sexual and apomictic reads mapping onto each *N13* family member was compared by using EdgeR (see “Materials and methods”). We found five *N13*-like isotigs aligning a significantly higher number of small RNAs in sexual samples (Table 3). The rest of the isotigs were equally represented in the sexual and apomictic libraries. These results indicated that, while all members of the *N13* family produce 24-nucleotide sRNAs, a few of them generate a higher number of small sequences in flowers of sexual plants with respect to apomictic ones.

**Table 3:** *N13*-like sequences showing differential small RNA representation in apomictic and sexual *P. notatum* florets

Isotig (global assembly)^a^	Apoisotig^b^ (accession number)	Sexisotig^b^ (accession number)	FDR^c^	Predicted target (*Zea mays*)	Target annotation
global24592	apoisotig10911 (GFMI02010965.1)	sexisotig09641 (GFNR01009661.1)	0.01798	ABQ44355.1 *Zea mays*	Polyprotein
global43969	apoisotig00491/apoisotig00493/apoisotig00495 (GFMI02000547.1/GFMI02000549.1/GFMI02000551.1)	sexisotig36836 (GFNR01036847.1)	0.01871	–	–
global51305	apoisotig15855 (GFMI02015904.1)	sexisotig32318 (GFNR01032333.1)	0.01223	–	–
global00522	apoisotig03642 (GFMI02003695.1)	sexisotig05141 (GFNR01005166.1)	0.03264	XP_008659471.1 *Zea mays*	F-box protein/BED zinc finger/DUF659 domain (unknown function)
global26542	apoisotig15855 (GFMI02015904.1)	apoisotig21181 (GFNR01021200.1)	0.07836	EYU25918.1 *Erythrante guttata*	Full ribosomal 40S protein SA. Ribosomal protein S2

^a^Identification number of the isotig [454 global (apo + sex) assembly] (Ortiz et al. 2017), presenting differential representation of sRNAs in the small RNA Illumina libraries originated from sexual and apomictic plants (NCBI SRA SRP099144)

^b^Identification number of the most related apomictic or sexual isotigs (454 apo assembly or 454 sex assembly) (Ortiz et al. 2017). The Transcriptome Shotgun Assembly (TSA) projects corresponding to the apomictic (Q4117) and sexual (C4-4x) samples have been deposited at DDBJ/ENA/GenBank under the accessions GFMI00000000 and GFNR00000000, respectively. The versions described in this paper are the first ones (GFMI02000000 and GFNR01000000, respectively)

^c^ *FDR* false discovery rate, i.e. the proportion of discoveries that are false among all discoveries

### *N13* displays similarity with lncRNAs

A BLAST search in the GreenC database of plant non-coding RNAs (Paytuví Gallart et al. 2016) revealed homology with *Medicago truncatula* lncRNA Medtr7g096390 (E-value: 0.00244976). Further surveys in the CANTATA plant lncRNA database (Szcześniak et al. 2016) showed similarity to *Oryza sativa* lncRNA CNT0026163, which is potentially involved in splicing regulation (E-value: 0.0022). Moreover, other five lncRNA sequences showed homologies at E-value: 0.086. A BLAST search in the NONCODE database (http://www.noncode.org/index.php) scored matches with 28 different lncRNAs. The similarity detection occurred always at the predicted recognition site. These observations led us to hypothesize that N13 is an lncRNA, which could be involved in the modulation of its target transcript splicing through an unknown mechanism. Based on this and former evidence, we re-named the N13 sequence as PN_LNC_N13, after *P. notatum* long non-coding sequence N13.

### The putative *PN_LNC_N13* target is expressed in *Paspalum notatum* reproductive organs

**Fig. 3:**
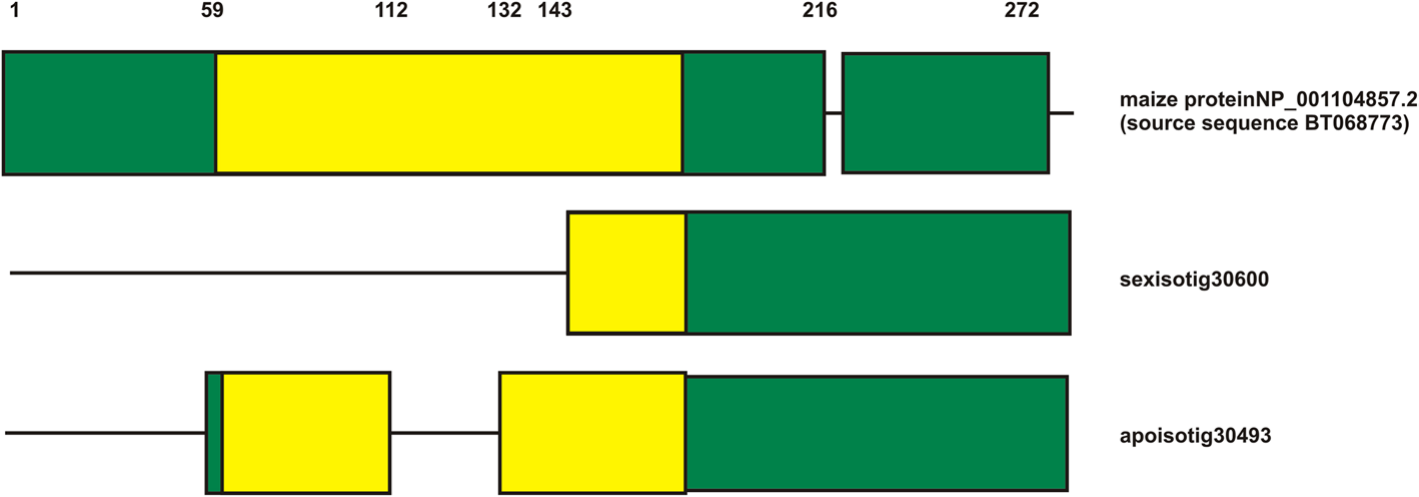
Alignment scheme for the putative *N13TAR* protein sequences originated from maize and *P. notatum*. Translation predictions made with the “ATG + alternative initiation codons”. The REC response receiver domain (complete for the maize and the apo isoforms) was marked in yellow. The alignment of the derived proteins is shown in Supplementary Online Resource 3

Next, we searched the sequences of the *PN_LNC_N13* putative target (*N13TAR*) in the assemblies of the 454/Roche FLX + floral RNA libraries of apomictic and sexual *P. notatum* (DDBJ/ENA/GenBank, accessions GFMI00000000 and GFNR00000000 for the apomictic and sexual libraries, respectively). The whole cDNA sequence from the predicted *N13* maize target (BT068773) was used as query. BLASTx searches followed by further clustal alignments and derived cladograms revealed that the predicted target most related sequences were apoisotig30493 (accession number GFMI02030540.1) (E-value: 3e^−82^; ID: 77%; Positives: 81%; Gaps: 2%) and sexisotig30600 (accession number GFNR01030615.1) (E-value: 4e^−82^; ID: 77%; Positives: 81%; Gaps: 5%). Proteins encoded by the *Paspalum* sequences were predicted for the 6 possible reading frames. A single possible ORF of significant size was detected for each of them. The predicted *Paspalum* protein sequences were 59 and 143 aminoacids shorter than maize BT068773 for the apomictic and the sexual isoforms, respectively (Fig. 3, Supplementary online resources 2 and 3). However, when using the “ATG and alternative initiation codons” option at ORF Finder, the predicted sexual protein started in a CTG alternative initiation codon and resulted only 12 aminoacids shorter than the apomictic isoform (71 aa shorter than the maize isoform) (Supplementary online resource 2). This observation indicated that either the sexual *Paspalum* sequence is significantly shorter than the apomictic one or it is translated from an alternative codon. Both *P. notatum* sequences (apomictic and sexual) lack the putative *N13* recognition site, which is located precisely at the 5′-terminal region in the maize long sequence (Supplementary online resource 2). An additional BLASTP search in the NCBI database using N13TAR protein sequences derived from apoisotig30493 and sexisotig30600 as queries revealed the existence of analogous shorter protein variants also in maize, namely RR10—Corn type-A response regulator [*Zea mays*] Sequence ID: ACG32848.1 (E-val: 3e^−109^, ID: 79%) and PREDICTED: two-component response regulator ARR9-like [*Zea mays*] Sequence ID: XP_008680710.1 (E-val: 1e^−43^; ID: 63%).

### *N13TAR* annotation

The maize BT068773 gene encodes RESPONSE REGULATOR 6, which is involved in plant hormone signal transduction. The complete sequence of *Paspalum N13TAR* (apoisotig30493) was used to identify the most likely *Arabidopsis* orthologue in BLASTX searches at TAIR. The best match was AT3G57040, which encodes ARR9, a two-component response regulator-like protein (length of the similarity region: 195 aminoacids (83.3%); E value: 9 e^−51^; ID: 59% (106/178); Positives: 71%; Gaps: 10%). Several ontology classes are associated with this sequence, like circadian rhythm, cytokinin-activated signaling pathways, phosphorelay signal transduction system, regulation of transcription and response to cytokinin. This regulator protein has a receiver (REC) domain, with a conserved aspartate residue and a possible phosphorylation site located at the N-terminal end. Moreover, it appears to interact with histidine kinase-like genes ATHP3 and ATHP2. The *P. notatum* apomictic isoform N-terminal end contains the complete REC signal receiver domain. In the sexual isoform predicted from the “ATG initiation codon” option, this functional region is cut in half, and the N-terminal phosphorylation site disappears (Fig. 3, Supplementary online resource 3). The rest of the protein is conserved, but for the inclusion of a small insertion of 4 amino acids at position 216 (reference: maize target sequence BT068773.1) and 4 additional amino acids at the C-terminal end, occurring in both *Paspalum* types (apo and sex) (Supplementary Online Resource 3). However, if the sexual isoform is translated from the alternative CTG codon, the REC signal receiver domain remains complete. The presence of shorter forms of the target protein in *Paspalum* flowers, the potential existence of different isoforms in apomictic and sexual plants and the location of the *N13* targeting site within the sequence region absent in the shorter forms, led us to hypothesize that the differential activity of *PN_LNC_N13* could be promoting the generation of transcript variants.

### Expression of *PN_LNC_N13* and *N13TAR* during reproductive development

**Fig. 4:**
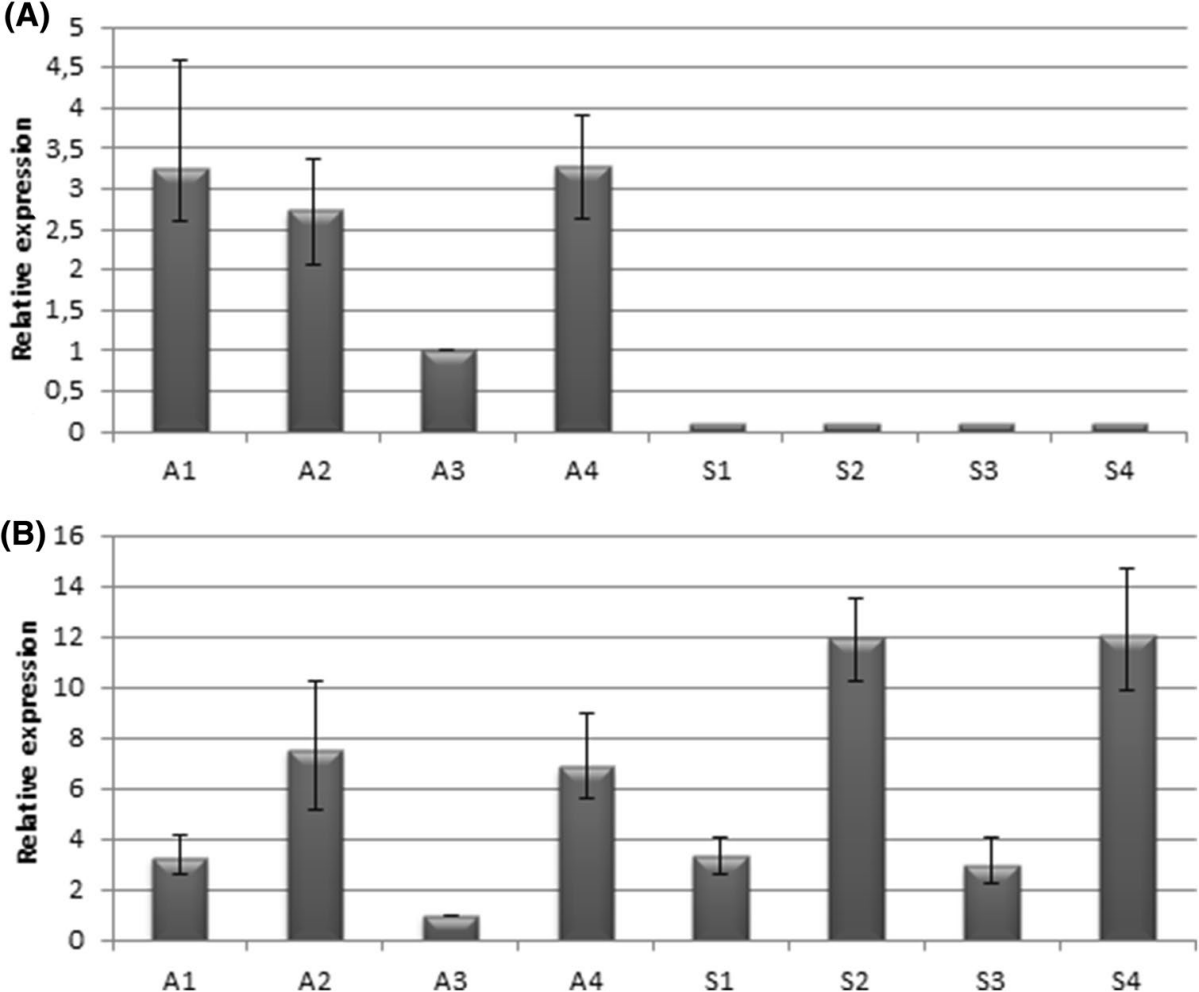
qPCR experiments reveal *N13*/*N13TAR* differential expression patterns between apomictic and sexual genotypes. **a** *PN_LNC_N13* is expressed only in apomictic genotypes and not in sexual ones, showing peaks at premeiosis, meiosis and anthesis. **b** *N13TAR* has similar expression profiles in both genotypes with upregulation in meiosis and anthesis, but it shows higher levels of representation in sexual genotypes. *A1* Apomictic genotype, premeiosis stage, *A2* Apomictic genotype, meiosis stage, *A3* Apomictic genotype, postmeiosis stage, *A4* Apomictic genotype, anthesis stage, *S1* Sexual genotype, premeiosis stage, *S2* Sexual genotype, meiosis stage, *S3* Sexual genotype, postmeiosis stage, *S4* Sexual genotype, anthesis stage

The expression of *PN_LNC_N13* was studied by qPCR in flowers of sexual and apomictic plants, along the *P. notatum* reproductive pathway. We tested if the primers had potential to produce multiple amplicons through an *in silico* PCR on the 60 genomic sequences homologous to *N13* (http://insilico.ehu.es/user_seqs/) (San Millán et al. 2013). Only the original *PN_LNC_N13* sequence was amplified *in silico*. Moreover, eight clones derived from Real-Time PCR amplifications of apomictic floral cDNA samples were sequenced by Macrogen (Korea) and produced sequences highly similar to the original *PN_LNC_N13* sequence, probably representing allelic variants (147 nt, query cover 100%; Expect: 0.0; ID: 92–96%; Gaps: 0%). Contrarily, eight clones derived from real time PCR amplifications of sexual floral cDNA samples generated shorter sequences (67 nt) composed mainly by the primers (probably derived from primer dimerization or amplification of variants in which primers were located at a very short distance, separated only by a few bases). Our results indicated that primers used for qPCR assays have the capacity to amplify specifically the *PN_LNC_N13* sequence and confirm that this transcript is not present in sexual flowers. Relative expression was evaluated in apomictic samples at premeiosis, late premeiosis/meiosis, post-meiosis and anthesis (Fig. 4), with upregulation detected at premeiosis, meiosis and anthesis.

The predicted target *N13TAR* also showed increased expression at meiosis and anthesis for both plant types. Although levels of expression are slightly higher in the sexual genotype, the general expression profile looks similar. Moreover, no significant quantitative differences in the number of reads were detected for *N13TAR* in the 454 sexual and apomictic libraries. Our results suggest that the expression of *PN_LNC_N13* is correlated only with a slight decrease in the quantitative level of expression of *N13TAR*, but the expression profile of the target seems unaltered. We also checked the presence of small RNAs associated with *N13TAR* sequences in the floral sRNA libraries generated from sexual and apomictic genotypes. No small RNAs were associated with them, neither in the sexual nor in the apomictic libraries. Here again, our results refute the existence of a silencing mechanism operating in the *N13* regulation activity.

### In situ hybridization analysis

**Fig. 5:**
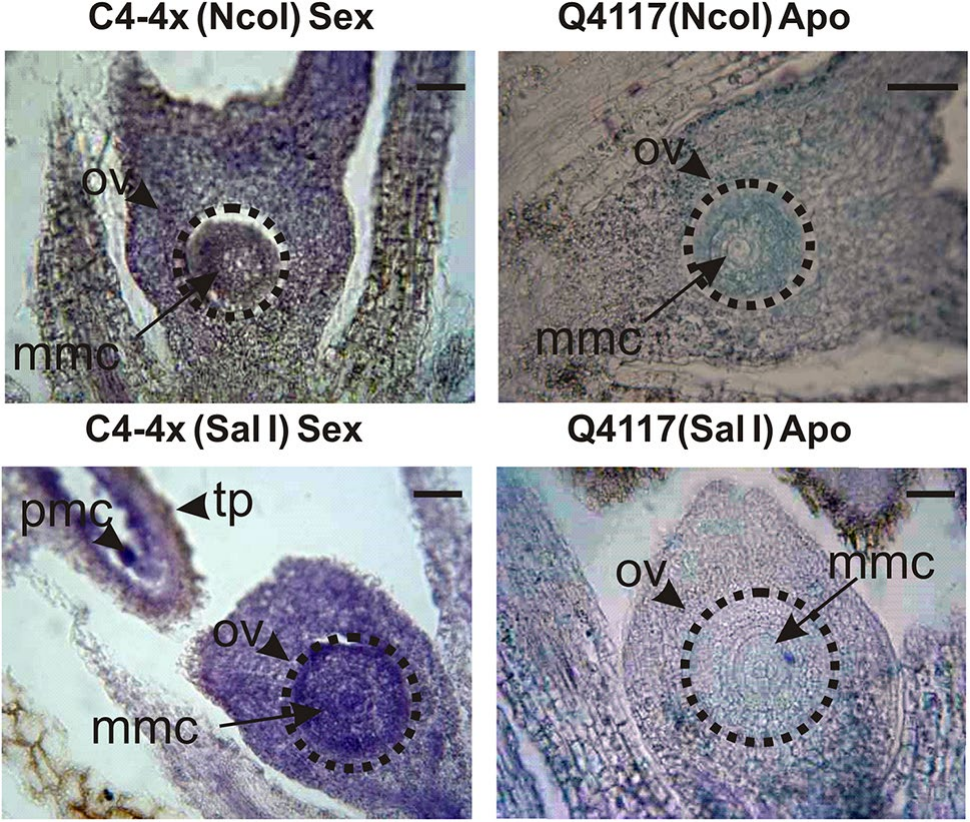
In situ RNA hybridization experiments in apomictic and sexual genotypes. The *Nco*I probe correspond to the (−) sequence, detecting the (+) strand. The *Sal*I probe corresponds to the (+) sequence, detecting the (−) strand. Both the *N13* (+) and (−) strands showed moderate to strong signals in nucella and pollen mother cells. A faint expression was observed in the Q4117 apomictic genotype. *Ov* ovule, *mmc* megaspore mother cell, *pmc* pollen mother cell, *tp* tapetum

We were interested in exploring the *in situ* distribution pattern at late premeiosis, since this is the onset time for apospory initials (i.e. the cells that give rise to unreduced embryo sacs, AI). Contrarily to qPCR primers, which were fully specific for *PN_LNC_N13*, the probes used in this experiment (the complete *N13* sequence, both + and − strands, 532 bp) can potentially detect all members of the *N13* family expressed in flowers (%ID ranging from 83 to 88%). Hybridization with a *PN_LNC_N13*-specific probe is unattainable, even after sub-cloning, since similarity with several different members of the family occurs all along the sequence (this is evident by doing a BLASTn search at NCBI using the *N13* sequence as query onto the TSA database, limited by *Paspalum notatum* taxid:147272). Therefore, in this experiment we expect that both long and small RNAs corresponding to at least several members of the *N13* family will hybridize. Results showed that both the sense and antisense probes gave rise to strong signals in ovules and anthers of the sexual genotype, but only faint ones were seen in apomictic ovules (Fig. 5). These results suggest that both strands (+ and −) of one or more family members are up-regulated in ovules of sexual plants at premeiosis, which is in agreement with the detection of an increased processing of sRNA in sexual plants for at least five members of the family (Table 2).

## Discussion

Natural apomictic plants are highly heterozygous polyploids, with large uncharacterized genomes plagued with repetitive elements, a condition that complicates molecular research in this field (Ozias-Akins 2006). Moreover, in the best characterized apomictic species the trait is controlled by large non-recombinant heterochromatic regions of 30–60 Mbp, where hundreds of candidate genes are organized as a single supergene, which is inherited as a chromosomal block (Akiyama et al. 2005; Pupilli et al. 2004; Stein et al. 2007). The difficulties implied in dissecting the components governing the trait from this complex region can be anticipated (Hand and Koltunow 2014). Notwithstanding these many drawbacks, impressive progress was achieved in the last few years. Apomictic species of the genera *Pennisetum, Poa, Paspalum, Hieracium, Eragrostis, Brachiaria* and *Boechera*, among others, were fully mapped and/or their floral transcriptomes and/or sRNA pools were fully characterized by using NGS (reviewed in Hand and Koltunow 2014). Although the first protein-coding genes involved in apomixis, *BABYBOOM* was recently characterized (Conner et al. 2015), the analysis of many protein-coding candidates remains pending, and the genetic determinants triggering the trait were still not identified. Moreover, a significant proportion of the transcripts differentially expressed between sexual and apomictic plants seem to correspond to ncRNAs as *ORC3* in *Paspalum* (Siena et al. 2016). Some of these candidates display partial homology with retrotransposons, but carry transduplicated gene sequences homologous to apomixis-associated functional genes, as was reported by Ochogavía et al. (2011) and Podio et al. (2014).

The mechanism underlying the function of long non-coding RNAs (lncRNAs) is a stimulating area of research, maybe because the work in this field is in its infancy and, in particular, characterization of the functional routes operating in plants is far behind that in humans and animals (Liu et al. 2015). In this work, we began the study of a *Paspalum* apomixis-associated ncRNA, namely *N13*. It represents a difficult task, since scarce molecular tools are available for apomictic species. The recent development in our laboratory of full genomic sequence raw data (unpublished) as well as long and short RNA databases originated from flowers of sexual and apomictic individuals was of great help. However, a particular aspect that complicates functional analysis is that ncRNAs are poorly conserved among different species. Therefore, genomic information and mutant collections generated from model plants may not be fully exploited. In addition, ncRNAs are usually expressed at very low levels, making them to look like transcriptional noise (Chekanova 2015).

Here we determined that the *PN_LNC_N13* family is composed by numerous different homologues (at least 60) without coding capacity, but carrying small stretches of protein-coding genes (predicted recognition sites). A few of these sequences are expressed at moderate levels in florets of both sexual and apomictic plants. Moreover, our data suggest that these sequences likely generate 24-nucleotide small RNAs from regions different from the recognition site. *PN_LNC_N13* is represented in florets (ovules and anthers) of apomictic plants only, but at least five members of the family show a higher level of sRNA representation in sexual plants, revealing an increased activity in these biotypes. Moreover, probes recognizing the *PN_LNC_N13* family hybridize preferentially in ovules and anthers of sexual plants in comparison with apomictic plants. Besides, at least some members of the family are expressed in ± orientation or antisense transcript copies are formed from the activity of an RNA-dependent RNA polymerase pathway. Both sense/antisense hybridization is observed in in situ hybridized tissues and ± sRNAs are detected. Regarding the target genes, differential expression and occurrence of allelic variants of different size are observed in flowers of sexual and apomictic plants, with genetic differences involving the predicted recognition sites. No small RNAs were associated with the targets. In the particular case of representative member *PN_LNC_N13*, it is expressed only in apomictic florets, with peaks of activity at premeiosis/meiosis and anthesis. Its putative target gene, *ARR9*-like response regulator, shows different transcript variants in apomictic and sexual plants. Whether *PN_LNC_N13* is responsible for the control of mechanisms leading to the generation of these variants should be investigated in further work by silencing and overexpressing it and analyzing the effect on the representation of the different target isoforms.

Recently, a possible link between the switch from apomixis to sexuality and the regulation of alternative splicing in *Paspalum* was hypothesized by Siena et al. (2014). In this work, evidence was reported on the existence of two forms of gene *TGS1* (trimethylguanosine synthase 1) in plants: (1) a canonical short form present in all eukaryotes, composed mainly of a methyltransferase domain (*TGS1*); and (2) an long plant-specific homologous isoform, containing a 5′ terminal extension with a WW domain (*TGS1*-like). The latter isoform showed expression rates negatively correlated with the percentage of aposporous embryo sacs (Siena et al. 2014). In yeasts and animals, *TGS1* has a dual function. On one hand, through a methyltransferase domain and two binding domains, it promotes interactions with RNA and S-adenosyl-L-methionine, the methyl donor in the methyl transfer reaction (Zhu et al. 2001), and catalyzes the post-transcriptional conversion of 7-methylguanosine caps (m^7^G) into 2,2,7-trimethylguanosine (m_3_G); this reaction plays a central role in the biogenesis of sn(o)RNAs and, consequently, in spliceosome assembly and activity. On the other hand, *TGS1* is pivotal for transcriptional modulation in several contexts. It interacts with and co-localizes to the nucleus along with histone acetyl transferase (HAT)-containing transcriptional coactivators such as CBP/Ep300 and non-HAT-containing coactivators such as the Mediator subunit Med1 (PPAR binding protein; PBP/TRAP220/DRIP205) and PRIP (Zhu et al. 2001; Misra et al. 2002; Kornberg 2007). A major set of TGS1 targets consists of ribosomal RNAs and ribosomal protein genes, proteasome-related proteins, cytoskeletal proteins, and ERK2 cascade member genes. Interestingly, the *N13* family members show several targets related with the ontological classes affected by TGS1 (like ribosomal 40S protein SA, F-box protein, ARR9 response regulator). Therefore, a possible link between the function of splicing regulator *PNTGS1*-like and the lncRNA *N13* family should be further investigated in future work aimed at elucidating the switch from sexual to asexual reproduction. It could be hypothesized that, while *TGS1* modulates the differential activity of the splicing machinery, *N13* family members point to specific targets that must be processed by alternative splicing.

A classification of *PN_LNC_N13* onto an lncRNA subclass would require additional information, which is still not available. According to Ma et al. (2013), lncRNAs are classified primarily based on major features like genomic location, functions exerted on DNA or RNA, functioning mechanisms and targeting mechanisms. The first aspect defines four different classes: antisense transcripts (lncNATs), intronic lncRNAs, promoter lncRNAs and long intergenic ncRNAs (lincRNAs) (Ariel et al. 2015). An accurate classification of *N13* into one of these categories would require a more detailed characterization of the genomic region from where it was originated. However, a *P. notatum* assembled genomic sequence is still not available. It is possible that *N13* is a long intergenic ncRNAs (lincRNAs), but confirmation will require chromosomal walking analysis or availability of data derived from genome sequencing projects. A second feature to be considered is the function exerted on DNA or RNA. Evidence shown here (like similarity with lncRNA CNT0026163 and the existence of genetic variants of the target involving the recognition site) suggests that *PN_LNC_N13* could be influencing transcript processing, but the operational mechanism remains unknown. Some possibilities might be anticipated, based on previous literature: it might bind to an intronic area in order to avoid splicing, modulate the pool of a modified splicing factor or block the spliceosomal complex formation, as it was reported for other lncRNAs (Ma et al. 2013). However, the occurrence of another still non-characterized mechanism cannot be discarded. Given the plethora of lncRNA possible functions that have been reported for other candidates (Xie and Fan 2016; Wu et al. 2016), additional or combined activities should be considered.

The presence of small sequences of opposite orientations associated with the *PN_LNC_N13* suggests the existence of RNA:RNA or RNA:DNA pairing during the action mechanism or precursor degradation in order to produce a shorter mature targeting sequence. However, *N13* could be involved in many lncRNA functional processes categories like signal (response to stimuli in specific cell types), decoy (bind and titrate away a protein target), guide (bind proteins and then direct the localization of ribonucleoprotein complex to specific targets), scaffold (serve as central platforms to form ribonucleoprotein complexes) or RNA structure mediated interactions and protein linkers (Ma et al. 2013). Here again, a single targeting archetype may not be sufficient to fully describe one lncRNA and additional work is needed to elucidate the action mechanisms.

We are aware on the profusion of ncRNA elements that might be controlling different aspects of apomictic development and the challenge implied in the task of classifying them, due to the low conservation they exhibit with respect to sexual model species. Moreover, the study of such a complex biological trait cannot be restricted to the comparison with model organisms, in which it is absent. Advances in this area will require full characterization of the genomes of *P. notatum* and other apomictic species, as well as the establishment of effective techniques aimed at knocking-down these sequences and analyzing the consequent reproductive phenotypes. Moreover, it will require the development of promoters directing expression to specific cell types and the generation of biomarkers revealing the acquisition of a particular developmental fate. In the next few years, the generation of biological tools and databases pointing to a better characterization of these molecules will be crucial to apomixis research.

## Electronic supplementary material

Below is the link to the electronic supplementary material.

Supplementary material 1 (PDF 217 KB)

Supplementary material 2 (PDF 241 KB)

Supplementary material 3 (PDF 134 KB)
