# Supplementary material for: Structure, target-specificity and expression of PN_LNC_N13, a long non-coding RNA differentially expressed in apomictic and sexual Paspalum notatum
Source: Plant Mol Biol. 2017 Nov 8;96(1):53–67. doi: 10.1007/s11103-017-0679-4 (PMC5778186; doi:10.1007/s11103-017-0679-4)
Supplement: Supplementary file 1 — Supplementary material 1 (PDF 217 KB) [file 11103_2017_679_MOESM1_ESM.pdf]

Structure, target-specificity and expression of *PNLNCN13*, a non-coding RNA differentially expressed in apomictic and sexual *Paspalum notatum*

Plant Molecular Biology

Ana Ochogavía[1], Giulio Galla[2], Guillermo Seijo[3], Ana María González[3], Michele Bellucci[4], Fulvio Pupilli[4], Gianni Barcaccia[2], Emidio Albertini[5], Silvina Pessino[1]

[1] Instituto de Investigaciones en Ciencias Agrarias de Rosario (IICAR)-CONICET/Laboratorio de Biología Molecular, Facultad de Ciencias Agrarias, Universidad Nacional de Rosario, Parque Villarino, Zavalla, Provincia de Santa Fe, S2125ZAA, Argentina.

[2] Laboratory of Genetics and Genomics, BreedOmics c/o DAFNAE, University of Padova, Campus of Agripolis, Viale dell'Università, 1635020 Legnaro, Italy.

[3] Instituto de Botánica Nordeste, Consejo Nacional de Investigaciones Científicas y Técnicas, Sargento Cabral 2131, Corrientes, 3400, Argentina.

[4] CNR-Istituto di Bioscienze e BioRisorse, Consiglio Nazionale delle Ricerche, UOS of Perugia, 06128, Italy.

[5] University of Perugia, Department of Applied Biology, Faculty of AgricultureBorgo XX Giugno 7406121 Perugia, Italy.

pessino@arnet.com.ar, spessino@unr.edu.ar

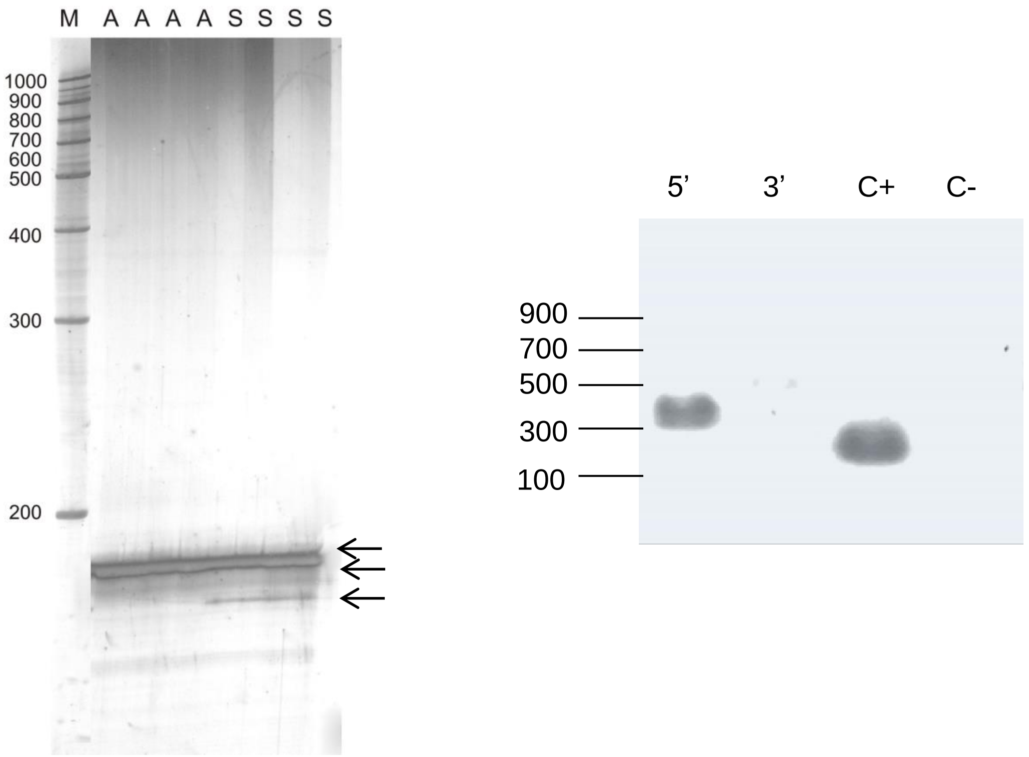

Supplementary Online Resource 1: Characterization of *N13* members. Left panel: In genomic amplifications, *N13* specific primers produced three major bands (indicated with arrows), one of which was present only in the sexual genotype. Four genomic DNA biological replicates of the same genotype (A: Q4117; S: Q4188) were used. Right panel: 5' and 3' RACE was conducted to extend the sequence. Only the apomictic sample 5' RACE reaction generated a band. C+: positive control (amplification was done with internal *N13* primers A13-upper-1 and A13-lower-2, see Table 1)
