# Supplementary material for: Structure, target-specificity and expression of PN_LNC_N13, a long non-coding RNA differentially expressed in apomictic and sexual Paspalum notatum
Source: Plant Mol Biol. 2017 Nov 8;96(1):53–67. doi: 10.1007/s11103-017-0679-4 (PMC5778186; doi:10.1007/s11103-017-0679-4)
Supplement: Supplementary file 2 — Supplementary material 2 (PDF 241 KB) [file 11103_2017_679_MOESM2_ESM.pdf]

Structure, target-specificity and expression of *PNLNCN13*, a non-coding RNA differentially expressed in apomictic and sexual *Paspalum notatum*

Plant Molecular Biology

Ana Ochogavía[1], Giulio Galla[2], Guillermo Seijo[3], Ana María González[3], Michele Bellucci[4], Fulvio Pupilli[4], Gianni Barcaccia[2], Emidio Albertini[5], Silvina Pessino[1]

[1] Instituto de Investigaciones en Ciencias Agrarias de Rosario (IICAR)-CONICET/Laboratorio de Biología Molecular, Facultad de Ciencias Agrarias, Universidad Nacional de Rosario, Parque Villarino, Zavalla, Provincia de Santa Fe, S2125ZAA, Argentina.
[2] Laboratory of Genetics and Genomics, BreedOmics c/o DAFNAE, University of Padova, Campus of Agripolis, Viale dell'Università, 1635020 Legnaro, Italy.
[3] Instituto de Botánica Nordeste, Consejo Nacional de Investigaciones Científicas y Técnicas, Sargento Cabral 2131, Corrientes, 3400, Argentina.
[4] CNR-Istituto di Bioscienze e BioRisorse, Consiglio Nazionale delle Ricerche, UOS of Perugia, 06128, Italy.
[5] University of Perugia, Department of Applied Biology, Faculty of AgricultureBorgo XX Giugno 7406121 Perugia, Italy.
pessino@arnet.com.ar, spessino@unr.edu.ar

```
BT068773maize      cagaaaaccacccccaagaactaaaaaactaaccgagccatatcatatgatatgatatga
apoisotig30493     ------------------------------------------------------------
sexisotig30600     ------------------------------------------------------------

BT068773maize      tgtgatatggtagtagcactagcagcagtatgaaaaaggcagagggaacaaaagaaaatg
apoisotig30493     ------------------------------------------------------------
sexisotig30600     ------------------------------------------------------------

BT068773maize      gtggtggatcctttcgggcccctgctctagaatactagcgcaactgatatggggtcaatt
apoisotig30493     ------------------------------------------------------------
sexisotig30600     ------------------------------------------------------------

BT068773maize      cgtggcaagatttggagcaaatctgctccaggatcttgctttccatgcgctgggactttc
apoisotig30493     ------------------------------------------------------------
sexisotig30600     ------------------------------------------------------------

BT068773maize      ttttagtcaccgcacctttttccccttcgctttgctttccacgattccacacatcatatc
apoisotig30493     ------------------------------------------------------------
sexisotig30600     ------------------------------------------------------------

BT068773maize      tcctcgaatcgtttcatgtgcttactgcttatatatatatatgtgtgtgtgtctgtcgac
apoisotig30493     ------------------------------------------------------------
sexisotig30600     ------------------------------------------------------------

BT068773maize      tcaagcacacacacacacatcctccaccaccattcatcagaaagcgaagcaccagcctct
apoisotig30493     ------------------------------------------------------------
sexisotig30600     ------------------------------------------------------------

BT068773maize      gatgccaagccaaagcacacacacctcctccagcctttcttcctctacaaccccaattct
apoisotig30493     -------------------gctatagcatccttctttccttccccataaggc---ataag
sexisotig30600     ------------------------------------------------------------
```

```
BT068773maize      tccctgcagctctgctgctgccttgtttcgatcggtcatggcggcggtggccacagagac
apoisotig30493     actgcacagctctg-gtctcttgctgcgtctgcggccatggcggcggtggccacgaagac
sexisotig30600     ----------------------------------------gtggcggtggccacggagac
                                                           * ************  ****

BT068773maize      tccgttccatgtcctggcggtggacgacagcctcccggacaggaagctcatcgagaggct
apoisotig30493     tcccttccatgtcctggctgtggatgacagcctcccagacaggaagctcatcgagaggct
sexisotig30600     tcccttccatgtcctggctgtggatgacagcctcccagacaggaagctcatcgagaggct
                   *** ************** ***** *********** ***********************

BT068773maize      cctcaagacctcttccttccaagtgaccactgtcgactccgggagcaaggcgctgcagtt
apoisotig30493     cctcaagacctcttccttccaagtaaccactgtcgattctggaagcaaggctctgcagtt
sexisotig30600     cctcaagacctcttccttccaagtaaccactgtcgattctggaagcaaggctctgcagtt
                   ************************ *********** ** ** ******** ********

BT068773maize      cctgggcctccatgaccaggacagcacggttcctcctgtccacacgcaccagctggatgt
apoisotig30493     cttggggatccatg----------------------------------------------
sexisotig30600     cttggggatccatg----------------------------------------------
                   * ****  ******

BT068773maize      ggctgccaatcaggatgtggctgtgaacctgatcatcacagactactgcatgcctggcat
apoisotig30493     --------------atgccgccgtcaacctcatcatcactgactactgcatgcctggcat
sexisotig30600     --------------atgccgccgtcaacctcatcatcactgactactgcatgcctggcat
                                 ***  ** ** ***** ******** ********************

BT068773maize      gacaggatatgacctgctcaagaagatcaaggagtcgtcgtctctcagagatatcccggt
apoisotig30493     gacgggatatgatctgctcaagaagattaaagaatcgtcgtctctcagagatatcccggt
sexisotig30600     gacgggatatgatctgctcaagaagattaaagaatcgtcgtctctcagagatatcccggt
                   *** ******** ************** ** ** **************************

BT068773maize      ggtgatcatgtcctctgagaacattccttcaaggatcaataggtgcctggaggaaggagc
apoisotig30493     ggtgatcatgtcgtccgagaacatcccttcaaggatcaataggtgcctggaggaaggagc
sexisotig30600     ggtgatcatgtcgtccgagaacatcccttcaaggatcaataggtgcctggaggaaggagc
                   ************ ** ******** ***********************************

BT068773maize      tgacgagttcttcctaaaacctgtgcggctatcagacatgaacaagctcaagccccacat
apoisotig30493     tgatgagttcttcctaaaaccagtgaggctatcagacatgagcaagctgaagccccacat
sexisotig30600     tgatgagttcttcctaaaaccggtgaggctatcagacatgagcaagctgaagccccacat
                   *** ***************** *** *************** ****** ***********

BT068773maize      actgaaaagcagatgcaaccaggaacagcaccagcaa------------agtgacagtca
apoisotig30493     actgaaaagcagatgcaaggagcactatcaccaggaacagcatcaccaaagtgacagaaa
sexisotig30600     actgaaaagcagatgcaaggagcactatcaccaggaacagcatcaccaaagtgacagaaa
                   ******************  ** *  * ****** **            ********  *

BT068773maize      cagtggcgaacgcaggaaccccaca---atcagcagcagcgatagcataaacaaccgcaa
apoisotig30493     cagtgatgaacgtggtaaccccgcaaacagcagcagcagcgatagcatcaacacccgcaa
sexisotig30600     cagtgatgaacgtggtaaccccgcaaacagcagcagcagcgatagcatcaacacccgcaa
                   *****  *****  * ****** **   * ****************** **** ******

BT068773maize      gagaaagggtgcaggcaccgaagaaatcttgccccagctggcaaacagatcaaggcacag
apoisotig30493     gagaaaggcagcagacaacgaagaaattttgccccag---acaaacagatcaaggcacag
sexisotig30600     gagaaaggcagcagacaacgaagaaattttgccccag---gcaaacagatcaaggcacag
                   ********  **** ** ********* *********    *******************

BT068773maize      ttaactgagaactgactagtacagctaaaaccttttctttttcact--------------
apoisotig30493     ttggaagcaaactgattagtatagctaaccccttaaaagttttttttcgctttccctggtt
sexisotig30600     ttggaagcaaactgattagtatagctaacccttaaaagtttttttcgctttcccctggtt
                   **    *  ****** ***** ******  * *      ***

BT068773maize      ---------ttactttggcttactgatttgtaacgtagatgttagccctggatttgcaaa
apoisotig30493     -cttgccttagtttttagcctactaatttgtaacacagatgttagccttgatttggaaaa
sexisotig30600     ctttgccttagtttttagcctactaatttgtaacacagatgttagcccttgatttggaaa
                                *** ** **** *********  *********** *   ** * ***

BT068773maize      acggaacggaatctgtaaactgatactgcttgagtcgaatcgatcgaagtgattctgc
apoisotig30493     tattaatggagcccataaacagagtgaccacatttcagctgaaa----------atg-
sexisotig30600     atataat---------------------------------------------------
                       **
```

Supplementary Online resource 2: Alignments between putative *N13TAR* sequences originated from maize and *P. notatum*. Both *Paspalum* sequences are shorter than the maize one. None of the floral *Paspalum* sequences displays the *N13* predicted recognition sites (marked in green). Translation predictions made with the “ATG initiation codon” option (initial ATG marked in red) show differences in the N-terminal extension among the three isoforms. Translation predictions made with the “ATG + alternative initiation codons” show that the sexual isoform could be translated from an alternative site (initial codon CTG, marked in magenta), producing a protein only 12 aa shorter than the apomictic isoform. The predicted stop codon was marked in light blue. The alignment of the derived proteins is shown in Supplementary Online Resource 3.
