# Supplementary material for: Structure, target-specificity and expression of PN_LNC_N13, a long non-coding RNA differentially expressed in apomictic and sexual Paspalum notatum
Source: Plant Mol Biol. 2017 Nov 8;96(1):53–67. doi: 10.1007/s11103-017-0679-4 (PMC5778186; doi:10.1007/s11103-017-0679-4)
Supplement: Supplementary file 3 — Supplementary material 3 (PDF 134 KB) [file 11103_2017_679_MOESM3_ESM.pdf]

Structure, target-specificity and expression of *PNLNCN13*, a non-coding RNA differentially expressed in apomictic and sexual *Paspalum notatum*

Plant Molecular Biology

Ana Ochogavía[1], Giulio Galla[2], Guillermo Seijo[3], Ana María González[3], Michele Bellucci[4], Fulvio Pupilli[4], Gianni Barcaccia[2], Emidio Albertini[5], Silvina Pessino[1]

[1] Instituto de Investigaciones en Ciencias Agrarias de Rosario (IICAR)-CONICET/Laboratorio de Biología Molecular, Facultad de Ciencias Agrarias, Universidad Nacional de Rosario, Parque Villarino, Zavalla, Provincia de Santa Fe, S2125ZAA, Argentina.
[2] Laboratory of Genetics and Genomics, BreedOmics c/o DAFNAE, University of Padova, Campus of Agripolis, Viale dell'Università, 1635020 Legnaro, Italy.
[3] Instituto de Botánica Nordeste, Consejo Nacional de Investigaciones Científicas y Técnicas, Sargento Cabral 2131, Corrientes, 3400, Argentina.
[4] CNR-Istituto di Bioscienze e BioRisorse, Consiglio Nazionale delle Ricerche, UOS of Perugia, 06128, Italy.
[5] University of Perugia, Department of Applied Biology, Faculty of AgricultureBorgo XX Giugno 7406121 Perugia, Italy.
pessino@arnet.com.ar, spessino@unr.edu.ar

```
MAIZE              MCVCLSTQAHTHTSSTTIHQKAKHQPLMPSQSTHTSSSLSSSTTPILPCSSAAALFRSVM
sexisotig30600     ------------------------------------------------------------
apoisotig30493     -----------------------------------------------------------M


MAIZE              AAVATETPFHVLAVDDSLPDRKLIERLLKTSSFQVTTVDSGSKALQFLGLHDQDSTVPPV
sexisotig30600     ------------------------------------------------------------
apoisotig30493     AAVATKTPFHVLAVDDSLPDRKLIERLLKTSSFQVTTVDSGSKALQFLGIHD--------


MAIZE              HTHQLDVAANQDVAVNLIITDYCMPGMTGYDLLKKIKESSSLRDIPVVIMSSENIPSRIN
sexisotig30600     -----------------------MPGMTGYDLLKKIKESSSLRDIPVVIMSSENIPSRIN
apoisotig30493     ------------AAVNLIITDYCMPGMTGYDLLKKIKESSSLRDIPVVIMSSENIPSRIN
                                          *************************************

MAIZE              RCLEEGADEFFLKPVRLSDMNKLKPHILKSRCNQEQ----HQQSDSHSGERRNPTI-SSS
sexisotig30600     RCLEEGADEFFLKPVRLSDMSKLKPHILKSRCKEHYHQEQHHQSDRNSDERGNPANSSSS
apoisotig30493     RCLEEGADEFFLKPVRLSDMSKLKPHILKSRCKEHYHQEQHHQSDRNSDERGNPANSSSS
                   ********************.***********::.     *:*** .* ** **:  ***

MAIZE              DSINNRKRKGAGTEEILPQLANRSRHS-----
sexisotig30600     DSINTRKRKAADNEEILPQA-NRSRHSWKQTD
apoisotig30493     DSINTRKRKAADNEEILPQT-NRSRHSWKQTD
                   ****.****.* .******  ******
```

Supplementary Online Resource 3: Clustal alignment and scheme of maize and *Paspalum* N13TAR sequences. The *Paspalum* sequences are shorter that the maize ortholog and show differences in the N-terminal extension between the sexual and the apomictic genotypes.

.
